# Electrospray‐based sample preparation for high‐resolution protein structure elucidation by cryo‐EM

**DOI:** 10.1002/ctm2.70405

**Published:** 2025-08-10

**Authors:** Jingjin Fan, Zi Yang, Liwen Liang, Jia Wang, Hongwei Wang, Zheng Ouyang, Xiaoyu Zhou

**Affiliations:** ^1^ Department of Precision Instrument State Key Laboratory of Precision Measurement Technology and Instruments, Tsinghua University Beijing China; ^2^ Ministry of Education Key Laboratory of Protein Sciences Beijing Advanced Innovation Center for Structural Biology, School of Life Sciences, Tsinghua University Beijing China; ^3^ Tsinghua‐Peking Joint Center for Life Sciences Tsinghua University Beijing China; ^4^ School of Biomedical Engineering Hainan University Hainan China

**Keywords:** cryo‐EM, electrospray Ionisation, molecule medicine, sample preparation methods, structure‐based therapeutics

1

Understanding protein structures at the molecular level is of paramount importance in contemporary biomedicine.^1^ The correlation between variations in protein structures and their biological functions is vital to life processes and intricately linked to the emergence of numerous diseases.^2^ Revealing the three‐dimensional structures of proteins can assist the fundamental research for biology as well as for design of precision therapeutics, a field known as ‘structure‐based therapeutics’.^3^ This research approach facilitates the identification of effective targets^4^ and enables the creation of drugs that specifically target individual proteins. An in‐depth understanding of protein structures also provides deeper insights into disease mechanisms, fostering innovation in the development of novel vaccines and diagnostic tools. It serves as new pathways for the discovery of biomarkers, which can lead to earlier disease diagnosis and more effective clinical interventions.^5^

Cryo‐electron microscopy (Cryo‐EM) is a powerful tool for high‐resolution elucidation of protein structures.^6^ It lets researchers visualise proteins, nucleic acids, and large biomolecular complexes in their native status, offering detailed views of their molecular structures. The sample preparation process for Cryo‐EM involves rapid freezing of sample solution containing biomolecules into a thin layer of vitreous ice, which frequently introduces undesired interfacial interactions.^7^ The biomolecules tend to move to the interfaces between the water and air or substrate, which can result in conformational changes or partial denaturation. This also causes drop of the biomolecule that impedes accurate structure determination, as shown in Figure 1, since the images taken by the Cryo‐EM would be all for only limited views of the molecules.^7^, ^8^, ^9^ This issue is particularly pronounced for delicate or flexible proteins, making it challenging to obtain reliable results with high resolutions.^10^ Therefore, overcoming these problems due to the interfacial effects is pivotal for enhancing the application quality of using Cryo‐EM for protein structure elucidation.

**FIGURE 1:**
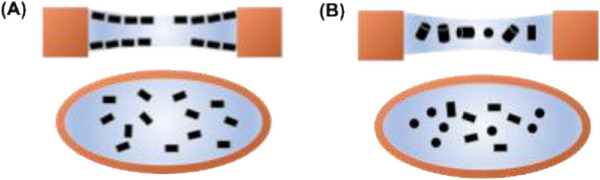
**Schematic illustration of the distributions of protein molecules under different conditions**. (A) Protein distribution in an uncharged droplet with undesirable preferred orientations due to the air–water interface effect, (B) protein molecules staying in the middle of a charged droplet with diverse orientations.

Significant efforts have been devoted to developing advanced cryo‐EM sample preparation methods, among which soft landing has emerged as an interdisciplinary and innovative one. Soft‐landing enables the deposition of ionic form of the molecules onto surface under controlled conditions, while preserving their molecular integrity and biological function.^11^, ^12^ Early demonstrations of the technique involved with organometallic and inorganic ions.^13^ With the advent of soft ionisation methods such as electrospray ionisation (ESI), soft‐landing of fragile biomolecules such as proteins has been achieved.^14^ The soft‐landed proteins could be analysed by traditional structural biology tools, such as EM,^15^ low‐energy electron holography,^16^ and cryo‐EM.^17^, ^18^

Recently, a new approach for preparing Cyo‐EM samples (ESI‐cryoPrep) was reported with cross‐disciplinary efforts,^19^ aiming to address the persistent issue of interfacial effects in Cryo‐EM, as shown in Figure 2. This method allows protein ions in electrified microdroplets to be softly deposited on Cryo‐EM grids in ambient atmosphere without significant damage or denaturation. The protein particles collected were found to be kept in the centre of the frozen liquid layer, thus overcoming the challenges posed by air–water interfaces during traditional sample preparation. It was believed that the charged small molecules inside a droplet were distributed at the gas–liquid interface while the charged large molecules, such as protein ions, were kept inside the droplet. Not only did this prevented the denaturing of the protein molecules but also allowed diverse orientations that are optimal for Cryo‐EM to produce high‐resolution images of the proteins.

**FIGURE 2:**
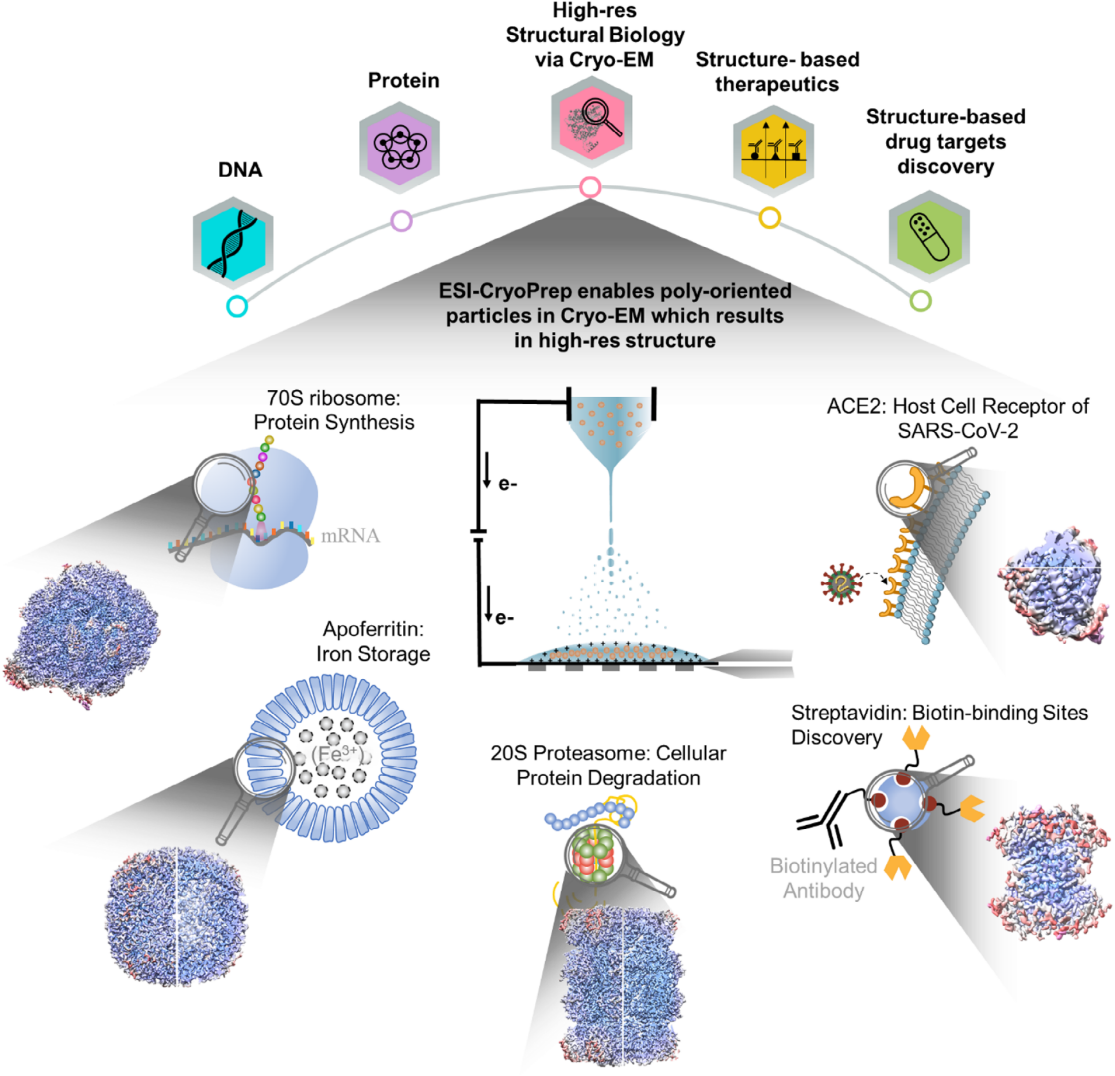
**High‐resolution protein structures elucidated by Cryo‐EM using ESI‐cryoPrep to prepare the samples,** including the 70S ribosome (protein synthesis), apoferritin (iron storage), 20S proteasome (protein degradation), streptavidin (biotin binding), and ACE2 (SARS‐CoV‐2 receptor). The structures of proteins are taken from Ref. (^19^), taken with permission from Springer Nature, ©2024.

The efficacy of this method has been demonstrated in a variety of structurally critical proteins that play essential roles in processes such as protein synthesis and degradation, as well as in the advancement of structure‐based therapeutics and drug discovery. In the case of the 70S ribosome, which is fundamental for protein synthesis, the method enabled researchers to capture its intricate, high‐resolution, and intact structure. This achievement holds potential for helping the development of novel antibiotics targeting bacterial ribosomes.^20^ For instance, apoferritin, a key protein involved in iron storage, was successfully analysed using this method, yielding high‐resolution images for its structural assembly. These insights have implications for understanding disorders associated with iron homeostasis.^21^ Similarly, the 20S proteasome, which is integral to cellular protein degradation, was visualised using this method, revealing its conformation. This conformational information is crucial for designing drugs that target proteasomal pathways, particularly for cancer treatment.^22^

Furthermore, this method has been instrumental in elucidating the detailed structure of streptavidin, a protein renowned for its robust binding affinity to biotin. This has significant implications for targeted drug delivery systems.^23^ By revealing the specific binding sites within the streptavidin‐biotin complex, Cryo‐EM has facilitated the development of biotin‐based drug carriers that can precisely deliver therapeutics to targeted tissues, thereby enhancing drug efficacy and minimising side effects

Additionally, Cryo‐EM played a pivotal role in the structural elucidation of the SARS‐CoV‐2 spike protein in complex with its human receptor, ACE2.^24^ This detailed structural understanding of the ACE2 binding site was essential for accelerating the development of antiviral drugs and neutralising antibodies aimed at preventing the virus from entering host cells. These examples underscore the indispensable role of Cryo‐EM in advancing structure‐guided drug design and addressing critical clinical challenges.

This soft‐landing of electrosprayed protein ions provides a simpler, more controlled method for preparing Cryo‐EM samples, particularly for maintaining diverse orientations of the proteins. The successful application of the ESI‐cryoPrep has shown promise in improving the resolution of protein structures, including the 70S ribosome (protein synthesis), apoferritin (iron storage), 20S proteasome (protein degradation), streptavidin (biotin binding), and ACE2 (SARS‐CoV‐2 receptor). By systematically addressing these diverse proteins, this approach showcases its broad applicability in structural biology, not only advancing our understanding of protein function but also driving innovations in precision medicine and targeted therapies.

## AUTHOR CONTRIBUTIONS

All authors contributed to writing of the letter.

## FUNDING

This project was funded by the National Key R&D Program of China (Grant Nos. 2023YFF0723500, 2024YFA1307300), National Natural Science Foundation of China (Grant Nos. 22374088, 22227807, 21934003, and 31825009), Major Project of Guangzhou National Laboratory (Grant No. GZNL2024A03004).

## CONFLICT OF INTEREST STATEMENT

The authors declare no conflicts of interest.

## ETHICS STATEMENT

This article does not contain any research involving humans or animals.
